# Respiratory syncytial virus among children hospitalized with severe acute respiratory infection in Kashmir, a temperate region in northern India

**DOI:** 10.7189/jogh.12.04050

**Published:** 2022-07-16

**Authors:** Parvaiz A Koul, Siddhartha Saha, Kaisar A Kaul, Hyder Mir, Varsha Potdar, Mandeep Chadha, Danielle Iuliano, Kathryn E Lafond, Renu B Lal, Anand Krishnan

**Affiliations:** 1 Sher-i-Kashmir Institute of Medical Sciences, Srinagar, India; 2 Influenza Program, US Centers for Disease Control and Prevention – Delhi office, India; 3 GB Pant Hospital for Children, Srinagar, India; 4 National Institute of Virology, Pune, India; 5 US Centers for Disease Control and Prevention, Atlanta, Georgia, USA; 6 All India Institute of Medical Sciences, New Delhi, India

## Abstract

**Background:**

Severe acute respiratory infections (SARI) are a leading cause of hospitalizations in children, especially due to viral pathogens. We studied the prevalence of respiratory viruses among children aged <5 years hospitalized with severe acute respiratory infections (SARI) in Kashmir, India.

**Methods:**

We conducted a prospective observational study in two tertiary care hospitals from October 2013 to September 2014, systematically enrolling two children aged <5 years with SARI per day. We defined SARI as history of fever or measured fever (≥38°C) and cough with onset in the last 7 days requiring hospitalization for children aged 3-59 months and as physician-diagnosed acute lower respiratory infection for children aged <3 months. Trained study staff screened children within 24 hours of hospitalization for SARI and collected clinical data and nasopharyngeal swabs from enrolled participants. We tested for respiratory syncytial virus (RSV) A and B, influenza viruses, rhinoviruses (HRV)/enteroviruses, adenovirus (AdV), bocavirus (BoV), human metapneumovirus (hMPV) A and B, coronaviruses (OC43, NL65, C229E), and parainfluenza viruses (PIV) 1, 2, 3 and 4 using standardized duplex real-time polymerase chain reaction.

**Results:**

Among 4548 respiratory illness admissions screened from October 2013 to September 2014, 1026 met the SARI case definition, and 412 were enrolled (ages = 5 days to 58 months; median = 12 months). Among enrolees, 256 (62%) were positive for any virus; RSV was the most commonly detected (n = 118, 29%) followed by HRV/enteroviruses (n = 88, 21%), PIVs (n = 31, 8%), influenza viruses (n = 18, 4%), BoV (n = 15, 4%), coronaviruses (n = 16, 4%), AdV (n = 14, 3%), and hMPV (n = 9, 2%). Fifty-four children had evidence of virus co-detection. Influenza-associated SARI was more common among children aged 1-5 years (14/18, 78%) while most RSV detections occurred in children <12 months (83/118, 70%). Of the RSV viruses typed (n = 116), the majority were type B (94, 80%). Phylogenetic analysis of G gene of RSV showed circulation of the BA9 genotype with 60bp nucleotide duplication.

**Conclusions:**

Respiratory viruses, especially RSV, contributed to a substantial proportion of SARI hospitalizations among children <5 years in north India. These data can help guide clinicians on appropriate treatment and prevention strategies.

Globally, respiratory viruses account for half of all causes of acute lower respiratory infections, including pneumonia and bronchiolitis [1,2]. Respiratory viruses have been detected in over half of children hospitalized with respiratory illness in studies from India and the US [3,4]. Among them, the respiratory syncytial virus is the leading cause of acute lower respiratory infection (ALRI) among children <5 years of age, frequently causing bronchiolitis and pneumonia in both high-income countries (HIC) as well as low- and middle-income countries (LMIC) [4-6]. Globally an estimated 33.8 million new episodes of RSV-associated ALRI occurred in 2005 and at least 3.4 million required hospitalisations [7]. High RSV-associated incidence of hospitalisation has been reported especially in LMICs [8,9]. Data from a community-based study in India showed annual incidence rates of 3.2/1000 among children <5 years of age [10].

As several candidate RSV vaccines are being evaluated in clinical trials, it is important to generate more data on RSV burden to inform future policy. This is crucial for India, as many of its states have populations comparable to large countries, but with wide variation between the states in terms of lower respiratory disease burden [11]. Various studies in the last decade have reported the prevalence of RSV among children hospitalized with ALRI from different parts of India with tropical climates, but none are reported from Kashmir, a temperate region of north India [12-15]. We present the prevalence of respiratory viruses among children aged <5 years hospitalized with severe acute respiratory infections in Kashmir.

## METHODS

### Study design

We prospectively enrolled children aged <5 years with severe acute respiratory infections (SARI) at two tertiary care hospitals from October 2013 to September 2014. We used the 2011 WHO case definition for SARI for children 3 months to 5 years, as an acute respiratory illness with history of fever or measured fever (≥38°C) and cough with onset within the last 7 days requiring hospitalization [16]. We considered children aged 8 days-3 months with physician-diagnosed acute lower respiratory infection as SARI irrespective of the signs and symptoms.

### Study setting

We conducted this study at two tertiary care public hospitals, Sher-i-Kashmir Institute of Medical Sciences (SKIMS) and Gobind Ballabh Pant Children’s Hospital (GBPCH) associated with Government Medical College; both are in Srinagar city (Latitude -34.0°N, Longitude 74.8°E) of Jammu and Kashmir in northern India. We selected these leading public hospitals as they serve most of the hospitalizations among children in Kashmir.

### Participant enrolment

We enrolled participants from October 2013 to September 2014. Trained study physicians visited the hospitals’ paediatric wards daily to screen children aged <5 years hospitalized in the past 24 hours for SARI. Children admitted with respiratory complaints were screened using a checklist to determine SARI eligibility. Among children who met the SARI case definition, we enrolled the first two SARI patients admitted in previous 24 hours. Written informed consent was given by the child’s parent or guardian prior to enrolment.

### Data and specimen collection

The study physician interviewed the parent/guardian and used hospital records to collect clinical data. Enrolled SARI patients were followed until discharge or death to record hospitalisation outcome. As these are very busy hospitals, case-sheets with all the symptoms and signs at admission were not always available; for consistency we collected caregiver reported symptoms (like “breathlessness”, “noisy breathing”) present at admission. Specimens were collected using nasopharyngeal swabs patients those enrolled within 24 hours of admission. The samples were transported the same day, in a viral transport medium (HiMedia Laboratories, Mumbai, India) and on ice to the laboratory.

Laboratory testing was conducted at the SKIMS influenza laboratory and the National Institute of Virology, Pune, India. Influenza testing was done using real-time reverse transcription polymerase chain reaction (RT-PCR) following CDC protocol [17]. Testing for other respiratory viruses was conducted using real-time RT-PCR assays for respiratory syncytial virus (RSV) A and B, para-influenza viruses 1-4 (PIV), human meta-pneumovirus (hMPV), corona viruses (NL63, OC43 and 229E), adenovirus (Adev), rhinovirus (HRV), rhinovirus/enterovirus and bocavirus, by in house standardized duplex real time qualitative RT-PCR (Applied Bio systems, USA) [18] using previously published primers and probes [19-24]. Testing for influenza viruses was completed on the same day of collection; however, deu to operational constraints, the other respiratory viruses were tested later using the frozen (-80°C) aliquots.

The expected amplicons were visualized on 2% agarose and purified using Charge switch magnetic beads PCR purification kit (Invitrogen^TM^, Thermo Fisher Scientific, Waltham, USA). DNA sequencing was carried out using Big Dye terminator V 3.1 cycle sequencing ready reaction kit (Applied Biosystems^TM^, Thermo Fisher Scientific, Waltham, USA) with M13 F and R primer, while unincorporated labelled ddNTP’s were purified using DyeEx 2.0 dye-terminated removal kit (Qiagen, Hilden, Germany). All specimens testing positive for RSV were sent to NIV for G gene sequencing and phylogenetic analysis. The sequencing was done on ABI 3730 DNA analyser; pair wise sequence alignment and protein translation was performed with Molecular Evolutionary Genetic Analysis (MEGA) version 6 program (www.megasoftware.net). Phylogenetic analysis of RSV detected in this study was done alongside clade-specific reference strain. MEGA version 6 was used for constructing neighbour-joining (NJ) trees using the Kimura’s two-parameter distance model, with 1000 bootstrap replicates.

### Data analysis

All data were entered in Epi-Info^TM^ (Centers for Disease Control and Prevention, Atlanta, US) and analysed using STATA 14/SE (StataCorp LLC, Texas, USA). We used bivariate analysis to compare the SARI cases tested positive for RSV with those testing negative for RSV and calculated odds ratios and *P*-values. We estimated adjusted odds ratio using multivariable logistic regression with detection of RSV as outcome variable using co-variates with *P*-value >0.1 in bivariate analysis (data not shown).

### Ethics

The institutional ethics committees/review boards of the Sher-i-Kashmir Institute of Medical Sciences, Srinagar, All India Institute of Medical Sciences, Delhi and US Centres for Disease Control and Prevention (CDC) approved the study. A written informed consent was obtained from the guardians of the children prior to enrolment.

## RESULTS

**Figure 1:**
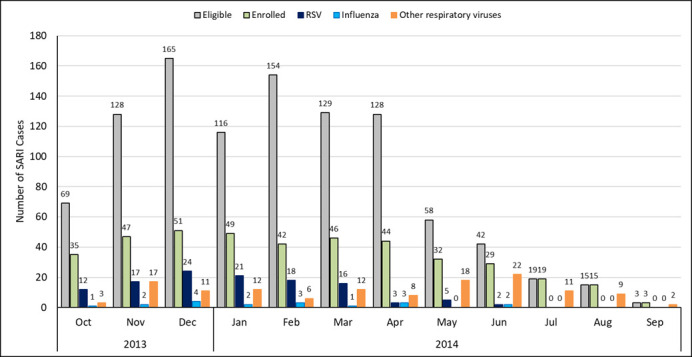
Month-wise distribution of viruses detected in children aged <5years hospitalized with severe acute respiratory illness (SARI) in Srinagar, India (2013-2014). The chart depicts the number of SARI cases by months during the project period. Columns coloured in grey indicate number of eligible cases who fulfilled the case definition; columns in light green indicate number of cases who were enrolled; columns in dark blue are number of enrolled cases tested positive for respiratory syncytial virus; columns in light blue indicate number of enrolled cases tested positive for influenza; columns in orange indicate the number of other respiratory viruses.

During the study period, there were 4548 paediatric admissions for respiratory illnesses in the study hospitals. Among these admissions, we found 1026 (22.6%) children aged <5 years who met the SARI case definition and were eligible for enrolment. We enrolled 412 (40.2%) children with SARI aged <5 years. The month-wise distribution of eligible and enrolled cases is shown in **Figure 1**. Nearly two-thirds (72.3%) of participants were <24 months and >50% were males (**Table 1**). Comorbidities were not common among children enrolled; asthma was reported most frequently reported (n = 24, 5.8%), followed by heart disease (n = 11, 2.7%), neurological conditions (n = 7, 1.7%), and malnutrition (n = 5, 1.2%).

**Table 1:** Demographic and clinical characteristics of children <5 years hospitalized with severe acute respiratory illness (SARI) in a tertiary care centre in Srinagar, India (2013-2014)

	All SARI	Any virus		RSV positive		Influenza positive	
	(n = 412)	(n = 256)	%*	(n = 118)	%*	(n = 18)	%*
**Age group**							
0-5 mo	105	77	73.3	46	43.8	2	1.9
6-11 mo	89	56	62.9	33	37.1	1	1.1
12-23 mo	102	52	51.0	16	15.7	10	9.8
≥24 mo	116	71	61.2	23	19.8	5	4.3
**Gender**							
Males	234	146	62.4	63	26.9	12	5.1
Females	178	110	61.8	55	30.9	6	3.4
**Underlying medical condition**							
Asthma	24	17	70.8	1	4.2	1	4.2
Heart disease	11	4	36.4	1	9.1	0	0.0
Neurological condition	7	3	42.9	1	14.3	0	0.0
Malnourished	5	4	80.0	1	20.0	0	0.0
**Household characteristic**							
Median No. of household members (IQR)	7 (5-10)	7	(5-10)	7	(5-10)	9.5	(6-12)
Median No. children in house (IQR)	3 (2-4)	3	(2-4)	3	(2-4)	3	(3-4)
No. of households >2.5 persons/room	98	64	65.3	29	29.6	5	5.1
Smoker member	283	176	62.2	86	30.4	14	4.9
History of recent ARI in family	158	89	56.3	46	29.1	5	3.2
**Cooking fuel used**							
LPG	314	194	61.8	92	29.3	16	5.1
Electricity	281	179	63.7	87	31.0	14	5.0
Kerosene	103	64	62.1	34	33.0	4	3.9
Biomass fuels (Firewood/charcoal/crop-waste/dung)	275	171	62.2	73	26.5	9	3.3
**Care seeking**							
Previous care sought	278	174	62.6	87	31.3	12	4.3
Median interval from onset to admission in days (IQR)	3 (2-4)	3	(2-4)	3	(2-4)	3	(2-4)
Median interval from onset to sampling in days (IQR)	4 (3-5)	4	(3-5)	4	(3-5)	4	(3-5)

mo – month, IQR – interquartile range

*The denominators for the proportions expressed in percentages are the number of SARI cases in the respective rows.

The median household size was 7 persons (inter-quartile range (IQR) = 5-9) with overcrowding (>2.5 persons per room) reported in the households of 23.8% of participants. The use of biomass fuels was very common for cooking (75.3%) and heating (100%). Over two-thirds of enrolled patients had one or more household members who were current smokers. The median time between onset and admission was 3 days (IQR = 2-4 days) and between onset and specimen collection was 4 days (IQR = 3-5 days).

**Figure 2:**
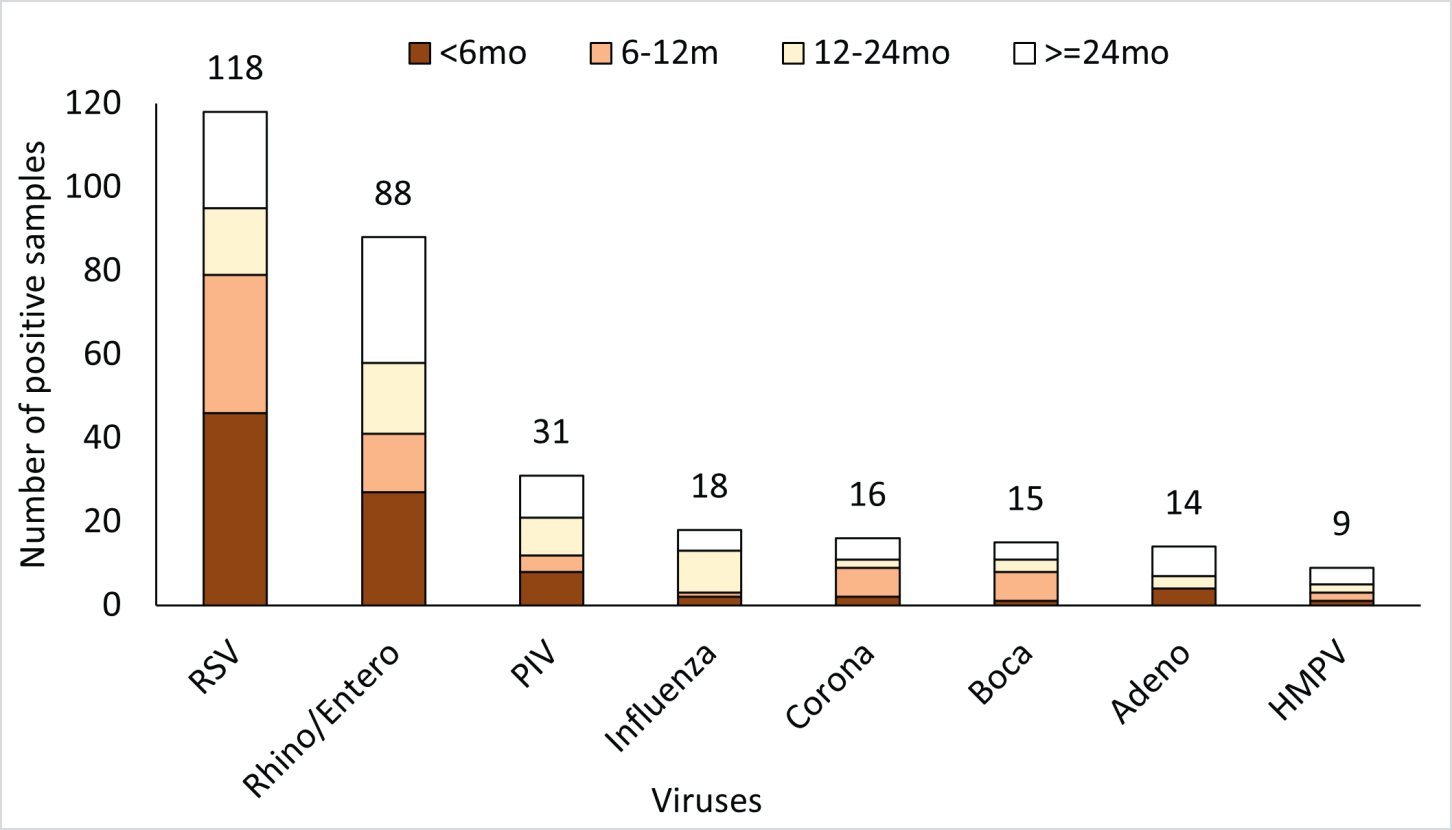
Age-group-wise distribution of viruses detected in children aged <5years hospitalized with severe acute respiratory illness (SARI) in Srinagar, India (2013-2014) The chart depicts the number of specimens testing positive for different respiratory viruses by age-groups. The dark red shaded area of the columns indicates the number of specimens collected from SARI cases aged <6months; lighter red area indicating age-group 6-12 months; lightest shade of red indicating age-group of 12-24 months; the white area indicating SARI cases aged ≥24 months. The viruses denoted here are arranged in order of their detection rate and include Respiratory Syncytial Virus, Rhinoviruses and Enteroviruses, parainfluenza viruses type 1-4, Influenza viruses, coronaviruses, bocavirus, adenovirus and human meta-pneumovirus (hMPV) The numbers at the top of each column represent the total number of specimens testing positive for the corresponding viruses.

We detected one or more respiratory viruses among 256 (62.1%; 95% CI = 57.2-66.8) enrolled SARI patients. Among these, RSV was most commonly detected (n = 118, 46.1%; 95% CI = 39.9-52.4) followed by HRV/enterovirus (n = 88, 34%; 95% CI = 28.6-40.5), PIV (n = 31, 12.1%; 95% CI = 8.4-16.7), influenza (n = 18, 7.0%; 95% CI = 4.2-10.9), coronaviruses (n = 16, 6.3%; 95% CI = 3.6-9.9), bocavirus (n = 15, 5.9%; 95% CI = 3.3-9.5), ADV (n = 14, 5.5%; 95% CI = 3.0-9.0), and hMPV (n = 9, 3.5%; 95% CI = 1.6-6.5) (**Figure 2**). Fifty-four patients had evidence of viral co-infections. Influenza-associated SARI was more common in children aged 1-5 years (83.4%; 95% CI = 58.6-96.4) compared with RSV infections, which were more common among children <24 months (80.5%; 95% CI = 72.2-87.2). RSV detection was nearly three times more likely among infants (adjusted OR = 2.6; 95% CI = 1.5-4.3) compared with children aged 2-5 years (**Table 2**).

**Table 2:** Demographic and clinical characteristics of children <5 years hospitalized with RSV positive compared with RSV negative cases of severe acute respiratory illness (SARI) in a tertiary care centre in Srinagar, India (2013-2014)

	RSV positive (n = 118)		RSV negative (n = 294)		Adjusted OR (95% CI)
	**n**	**%**	**n**	**%**	
**Demographic characteristics**					
Infants (age <1y)	80	67.8	128	43.1	**2.5 (1.5-4.3)**
Males	63	53.4	171	57.6	0.9 (0.5-1.4)
Tobacco smoker in household	86	72.9	197	66.3	1.0 (0.6-1.6)
Overcrowding (>2.5 persons/room)	29	24.6	69	23.2	1.0 (0.8-1.3)
Use of smoke-fuel	73	61.9	202	68.0	0.8 (0.4-1.3)
Breastfed (for infants <1y)	69	58.5	98	33.0	-
**Clinical and treatment history**					
ARI in family member	46	39.0	112	38.1	0.8 (0.5-1.4)
Asthma	1	0.8	23	7.8	0.1 (0.0-1.1)
Any heart disease	1	0.8	10	3.4	-
Any neurological disease	1	0.8	6	2.0	-
Received previous care before hospitalization	87	73.7	191	65.0	1.5 (0.9-2.5)
Delay in admission (>4d)	53	44.9	96	32.7	1.6 (0.8-2.9)
**Symptoms & signs**					
Cough	118	100.0	291	99.0	-
Fever	115	97.5	286	97.3	-
Breathlessness	105	89.0	249	84.7	-
Noisy breathing	103	87.3	243	82.7	**2.9 (1.3-6.2)**
Nasal discharge/congestion	92	78.0	226	76.9	
Diarrhoea	36	30.5	56	19.0	**1.8 (1.0-3.1)**
Sore throat	2	1.7	15	5.1	-
Earache	0	0.0	3	1.0	-
Rash	2	1.7	2	0.7	-
Tachypnoea	29	24.6	75	25.5	1.1 (0.7-1.9)
Wheeze	96	81.4	255	86.7	0.5 (0.2-1.0)
Any sign of respiratory distress*	49	41.5	159	54.1	0.6 (0.3-1.0)
Lethargy	71	60.2	191	65.0	1.1 (0.6-1.8)
**Laboratory detection**					
Detections of >1 virus	27	22.9	27	9.2	**4.6 (2.3-9.2)**

d – day, y – year

*Defined as presence of any of the signs of chest in-drawing, nasal flaring, cyanosis, stridor, head bobbing, accessory muscle use.

Besides cough (100%) and fever (97.5%) which were part of case-definition, the most common clinical symptoms among RSV-associated SARI patients were breathlessness (97.5%), noisy breathing (87.3%), and nasal discharge (78%). We found that noisy breathing (adjusted OR = 2.9; 95% CI = 1.3-6.2), and diarrhoea (adjusted OR = 1.8; 95% CI = 1.0-3.1) were more commonly reported among RSV-associated SARI patients compared with non-RSV SARI patients. RSV-associated SARI cases were more likely to have more than one viral detection (adjusted OR = 4.6; 95% CI = 2.3-9.2) compared to RSV negative SARI cases who tested positive for other respiratory viruses. The other viruses co-detected among RSV positive SARI cases were HRV (n = 14), PIV(n = 3), influenza (n = 2), seasonal corona viruses (n = 5), bocavirus (n = 3), and adenovirus (n = 3). The common clinical diagnoses for cases detected to have RSV were acute bronchiolitis (n = 45), pneumonia (n = 35), and lower respiratory infection with wheezing (n = 25). Antibiotics were used in 60.5% (95% CI = 55.6-65.3) of all enrolled SARI cases.

**Figure 3:**
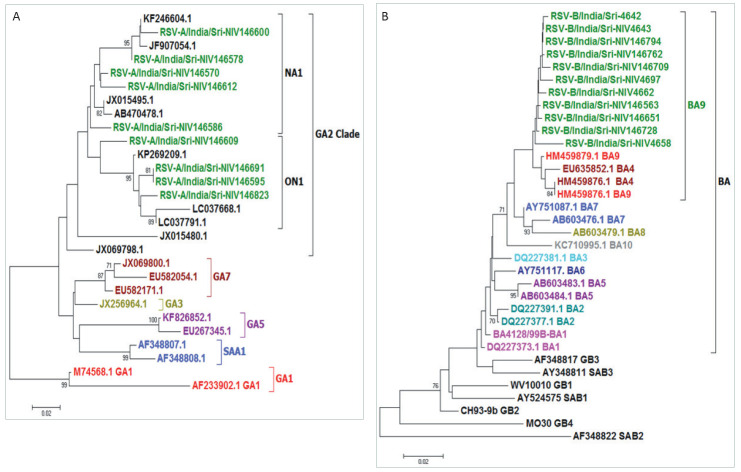
Phylogenetic analysis of G gene of RSV detected in upper respiratory specimens of children aged <5 years hospitalized with severe acute respiratory illness (SARI) in Srinagar, India (2013-14). Panel A is for RSV-A and Panel B is for RSV-B.

During the study period, over 80% of RSV detections were during months of October to March (**Figure 1**). Among the RSV positive specimens that were typed (n = 116), most were type B (n = 94, 81.0%; 95% CI = 72.7-87.7) and mostly detected in December 2013, while RSV-A (n = 22, 19.0%; 95% CI = 12.3-27.3) was mainly detected from October to November 2013. Phylogenetic analysis of G gene of RSV showed circulation of the BA9 genotype with 60 base-pair nucleotide duplication. (**Figure 3**).

Influenza was not commonly detected among SARI patients, and over 80% of influenza detections occurred from November to April. Influenza A(H3N2) virus was detected among 13 SARI patients (3.15%; 95% CI = 1.7-5.3) while influenza B was detected among 5 SARI patients (1.2%; 95% CI = 0.4-2.8).

## DISCUSSION

This is one of a few studies from India and the first study from a temperate north Indian region that examined and identified the presence of common respiratory viruses among children hospitalized with SARI using RT-PCR. Our study showed that nearly one-fourth of paediatric respiratory admissions were due to SARI among children aged <5 years.

More than half of the illnesses among children aged <5 years admitted with SARI in these hospitals were associated with respiratory viruses. These findings are similar to studies from Jaipur (60%) in western India [25]. RSV was most frequently detected among SARI patients; other pathogens detected were HRV, PIV, influenza, seasonal corona viruses, bocavirus, adenovirus, and hMPV. In this study, the burden of viral pathogens was higher among infants (<12 months old) who made up more than half of viral detections and was similar to studies elsewhere in India [26,27]. Moreover, similar to other studies in India and around the world, most of influenza-associated SARI patients were children aged 1 < 5 years [5,6,25,26,28].

RSV was the most common virus detected among the children with SARI, especially among infants, which was similar to other studies [12,14,29,30]. A recent meta-analysis [31] showed RSV as the most common cause of childhood ALRI and a major cause of hospital admission. It was shown that 99.0% of the global RSV-related deaths from 2000 to 2015 took place among resource-limited countries [31]. Moreover, India, China, Nigeria, Pakistan, and Indonesia together accounted for a total of 16 million cases of RSV infections and half of the global under-5 childhood deaths in the world from 2000 to 2015 [32].

Co-detection of other respiratory viruses was significantly higher among SARI cases positive for RSV, even after adjusting for other covariates in the multivariable analysis. Since the clinical and epidemiological significance of such co-detections are not well understood [33], increasing use of multi-pathogen assays might help generate more data to understand the etiological, clinical, and public health significance of such co-detections.

Our study was conducted over a one-year period and RSV was predominantly detected during winter months, from November to March. Because of its short duration, it is not possible to define seasonality of RSV circulation in this part of the country. Globally, it has been observed that RSV activity occurs between March and June in temperate regions of southern hemisphere (SH) and between September and December in the northern hemisphere (NH), with very low detections outside the season [34]. In tropical regions, RSV are detected more in late summer or rainy seasons [35]. In temperate climates, the activity correlates with decrease in temperature [34], which our study also observed. In north India, RSV mainly peaks in winter and some correlation with low temperature has been observed [26,36]. In Odisha in eastern India, RSV infections showed seasonal variation, with peaks during the rainy season followed by winter season [26]. It would be worthwhile to conduct a multi-site surveillance in tropical areas to assess the seasonality of RSV circulation to inform clinicians and policy makers.

The RSV subtyping showed presence of both RSV subtypes, but RSV type B predominated. Both RSV type A and type B have been reported from India [36-38]. BA-9 subtype was documented in our patients. Different genotypes of Group A (GA2, GA5, NA1 and ON1) and Group B (GB2, SAB4 and BA) have been described from India. BA genotype of type B has 60 bp duplication and ON1 genotype of type A has 72 bp duplication in the G gene.

Our study is not without limitations. We could not conduct this study beyond one year because of resource constraints. However, we were able to detect common respiratory viruses during this period and these coincided with expected timing of the circulation of these pathogens. We conducted this study in a setting where bad weather and city-wide curfews occasionaly interrupted data collection. This restricted the hospital visits by project staff and might have led to fewer admission on those days, which may have affected our ability to detect all circulating viruses in this region. However, the two study hospitals, Sher-i-Kashmir Institute of Medical Sciences (SKIMS) and Gobind Ballabh Pant Children’s Hospital (GBPCH), are the main public hospitals admitting patients from Srinagar and adjacent areas of Kashmir. Many children requiring admissions are likely to be brought in these two hospitals. We used the SARI case definition, so most RSV positive cases had fever (97.4%; 95% CI = 92.7-99.5); however, this symptom is considered a poor predictor of RSV [10]. Therefore, it is likely that we may have missed detection of RSV among children aged ≥3 months presenting with afebrile respiratory illness. This is a challenge for any surveillance platform for pan-respiratory viral pathogens and highlights one of the challenges of using surveillance data to estimate burden of disease. However, studies like ours suggest that, despite the limitations, SARI is likely to be an appropriate case-definition for detection of many common respiratory pathogens among children <5 years. The testing for non-influenza respiratory viruses was done at a later period of study using frozen aliquots, which might have affected the detection of these viruses. Despite these limitations, our study is the first from a northern temperate region of India to demonstrate the burden of SARI among children aged <5 years and the high frequency of RSV.

## CONCLUSIONS

Respiratory viruses, especially RSV, contributed to a substantial proportion of SARI hospitalizations among children <5 years in north India. These findings should help inform paediatricians and public health practitioners for appropriate management of SARI cases.
